# Observations on the Education of the Apothecary

**Published:** 1813-05

**Authors:** 


*To the Editors of the Medical and Physical Journal.*

GENTLEMEN,

I HAVE been much amused of late by reading the observations contained in your Journal on the subject of Apothecaries, upon which I have hitherto thought much without saying any thing; nor should I have deviated from those maxims now, had I not (to use a medical phrase) been stimulated to it by the illiberal remarks of Censorinus, in your 168th Number.

I have no wish to say any thing disrespectful of that part of the medical profession of which he is a member, though I cannot but differ in opinion with him; for I must beg to assure him, that I do not feel myself in the least offended at those expressions contained in his letter to Sir Francis Milman, though I must at the same time own that it would have been quite as decent had some of them been omitted. It appears very evident, if I am not greatly mistaken, that his own arguments most unequivocally point out the great necessity there is for those regulations, for the improvement of that class of practitioners in pharmacy termed Apothecaries, which the proposed bill is intended to produce; for, if they really are so illiterate as he describes them to be, surely it is high time they were better educated; it being well known that, in the country, at least nine-tenths of the community must depend solely upon this very ignorant class of the profession for medical aid; it therefore will scarcely admit of a doubt but that the public will be much benefited by it, unless it can be proved that ignorance is preferable to information. I certainly think the physician a very useful and valuable member of society; but, surely, it does not follow that the apothecary is of no use at all, and is tamely to submit to be trampled upon, and treated as a mere nonentity in matters of physic; for it will, I think, be admitted, that there are ignorant physicians as well as ignorant apothecaries. It is not for me to speak of the merits or demerits of that body of which I am an humble member; but I sincerely hope, whatever may be their defects, that they may be removed, to effect which I have contributed my mite; and, in my correspondence with Mr. Burrows upon the subject, I have been much pleased with his very liberal and disinterested sentiments, which certainly are as creditable to himself as they are serviceable to the cause of which he is the zealous advocate.

Perhaps Censorinus will be surprised when I inform him, that, in the county where I reside, there has scarcely been a physician employed for nearly half a century, (unless he admits that a Scotch diploma constitutes one, and which the "sleek and pampered apothecary" may at all times procure for a few pounds,) and that the sole regulation of a large infirmary, and nearly the whole practice of the county, medical and surgical, has been vested in the hands of a practitioner of this description for that space of time, notwithstanding various attempts have been made to oppose him by the "classical learning and medical erudition" of the universities of England and Scotland, and some of these were made by men of acknowledged medical acquirements; I therefore trust he will give merit its due, and allow that the knowledge of medicine is not entirely confined to the medical students of an university. I have not procured a diploma from the Royal College of Aberdeen, being convinced that I should not be one doit the wiser for it; nor do I ever mean to have one, unless I am obliged to have recourse to it upon the principle of self-defence; thinking it unjust to infringe upon the privileges of the graduate, and being perfectly satisfied with practising "surgery, pharmacy, and midwifery," provided I am allowed to make a reasonable charge for my attendance in lieu of drenching my patients stomachs with medicine, which is now, and has long been, the just opprobrium of medical science, and has led to those very salutary regulations that have been proposed to the legislature, and which I hope will be carried into effect, being well assured that the public will be much benefited by them.

I cannot but think the caution given to Sir Francis Milman and the College of Surgeons, against being "cajoled by the specious and sophistical arguments contained in the resolutions" of the Committee of Apothecaries, too contemptible to merit observation. I shall also pass over in silence those passages passages which savour more of self than philosophy; and merely add, that, if Censorinus and his brethren have done all in their power to starve the apothecaries by sending their prescriptions to the druggists, they have only to thank themselves for having made that rod, of which they now so loudly complain, and very justly anticipate the effects; and may therefore cease to wonder that this want of liberality on their part should cause so much jealousy and malevolence to exist between them and the apothecaries.

I confess I am one of those that claim the privilege of prescribing for the sick, although not much versed in the "language and doctrines of the Alcoran;" and shall not relinquish it, until I can discover some more substantial reason than is given by Censorinus why I should not.

If you think the above remarks worthy of a place in your useful and widely-circulated miscellany, I shall be obliged by their insertion. I have the honor to be, Gentlemen, very respectfully, Your obedient humble Servant,

*March* 8, 1813. MEDIOCRITAS.

